# Personal

**Published:** 1855-10

**Authors:** 


*Personal.*—Dr. H. D. Bulkley, for four years past one of the editors of the New York Medical Times, has retired from the tripod.

